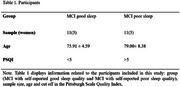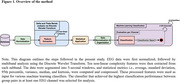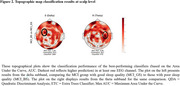# EEG low conventional bands non‐linear machine learning‐based analysis for Classifying MCI and sleep quality as a function of brain complexity

**DOI:** 10.1002/alz70861_108401

**Published:** 2025-12-23

**Authors:** Lucía Penalba‐Sánchez, Pedro Baptista Ribeiro, Mark Crook‐Rumsey, Alexander Sumich, Christina Howard, Saeid Sanei, Ahmad Zandbagleh, Hamed Azami, Emrah Düzel, Dorothea Hämmerer, Pedro Miguel Rodrigues

**Affiliations:** ^1^ German Center for Neurodegenerative Diseases (DZNE), Magdeburg Germany; ^2^ Institute of Cognitive Neurology and Dementia Research (IKND), Otto von Guericke University, Magdeburg Germany; ^3^ Centro de Biotecnologia e Química Fina (CBQF) Universidade Católica Portuguesa, Porto Portugal; ^4^ UK Dementia Research Institute Centre for Care Research and Technology, London UK; ^5^ Maurice Wohl Clinical Neuroscience Institute, King’s College London, London UK; ^6^ Nottingham Trent University, Nottingham UK; ^7^ Imperial College London, London UK; ^8^ Iran University of Science and Technology, Tehran, Tehran Iran (Islamic Republic of); ^9^ Centre for Addiction and Mental Health, Toronto, ON Canada; ^10^ University of Toronto, Toronto, ON Canada; ^11^ Center for Behavioral Brain Sciences, Magdeburg Germany; ^12^ Institute of Cognitive Neurology and Dementia Research (IKND), Otto‐von‐Guericke University, Magdeburg Germany; ^13^ University Hospital Magdeburg, Magdeburg Germany; ^14^ Institute of Cognitive Neuroscience, University College London (UCL), London UK; ^15^ Institute of Cognitive Neuroscience, University College London, London UK; ^16^ German Center for Neurodegenerative Diseases, Magdeburg Germany; ^17^ University of Innsbruck, Innsbruck, Tyrol Austria; ^18^ Institute of Cognitive Neurology and Dementia Research, Otto‐von‐Guericke University Magdeburg, Magdeburg Germany

## Abstract

**Background:**

Good sleep quality is essential for both physiological and mental health. It helps in clearing TAU and beta‐amyloid aggregates and consolidating memory, key processes in delaying dementia. Poor sleep is linked to reduced cognitive flexibility in daily life, likely due to decreased brain complexity, reflecting a reduced range of adaptive spatiotemporal brain dynamics. This study introduces a novel approach using non‐linear EEG analysis focused on low conventional bands to classify sleep quality in individuals with mild cognitive impairment (MCI), based on brain complexity.

**Method:**

Resting‐state EEG was collected from 22 participants with MCI aged 60+, grouped by sleep quality (Pittsburgh Sleep Quality Index): 11 MCI with good sleep, and 11 MCI with poor sleep (Table 1). EEG data (128 channels, 5‐minute recordings) were normalized and decomposed using the Discrete Wavelet Transform to reach delta (1–4 Hz) and theta (4–8 Hz) bands. Ten non‐linear complexity features, namely approximate entropy, correlation dimension, detrended fluctuation analysis, energy, Higuchi fractal dimension, Hurst exponent, Katz fractal dimension, Boltzmann Gibbs entropy, Lyapunov exponent and Shannon entropy, were extracted from 5 second segments. Statistical measures (mean, standard deviation, 95th percentile, variance, median, kurtosis) were computed from these time‐distribution features. These statistics were then used for training and testing a set of classic machine learning classifiers, employing leave‐one‐out cross‐validation (Figure 2).

**Results:**

Brain complexity successfully classified sleep quality in MCI, achieving an accuracy and area under the curve (AUC) of 1 in channel D13 (delta subband) using Quadratic Discriminant Analysis (QDA), and an accuracy of 0.94 and an AUC of 0.95 in channel B17 (theta subband) using the Extra Trees Classifier (ETC) (Figure 3).

**Conclusion:**

Specific machine learning classifiers distinguish excellently sleep quality in MCI using spatiotemporal complexity features from slow EEG subbands. The most relevant channels for group discrimination were primarily located in bilateral temporal regions of the neocortex known to be among the first affected in amnestic MCI, as previously shown in neuroimaging studies. Future longitudinal studies could investigate whether changes in brain complexity within these slow‐frequency temporal regions, influenced by sleep quality, are associated with an earlier or faster onset of dementia.